# Prolonged exposure therapy and supportive counselling for posttraumatic stress disorder in adolescents in a community-based sample, including experiences of stakeholders: study protocol for a comparative randomized controlled trial using task-shifting

**DOI:** 10.1186/s12888-018-1873-x

**Published:** 2018-09-06

**Authors:** Jaco Rossouw, Elna Yadin, Debra Alexander, Soraya Seedat

**Affiliations:** 1 Stellenbosch University, Stellenbosch, Western Cape South Africa; 2 Department of Psychiatry, University of Pennsylvania, Philadelphia, PA USA; 3 Centre for Cognitive-Behaviour Therapy, 67 Visagie Street, Monte Vista, Cape Town, Western Cape 7460 South Africa

**Keywords:** Treatment outcome, Posttraumatic stress disorder, Prolonged exposure, Supportive counselling, Randomized controlled trial, Task-shifting

## Abstract

**Background:**

There is a dearth of empirical evidence on the effectiveness of pharmacological and non-pharmacological treatments for adolescents with posttraumatic stress disorder (PTSD) in developing countries. The primary aim of the study is to examine the effects of prolonged exposure therapy compared with supportive counseling for adolescents with PTSD delivered by nurses trained as counselors.

**Methods/design:**

A single-blind randomized clinical trial comprising 90 adolescents with PTSD using a permuted block design will be utilized. Nurses previously naïve to prolonged exposure and supportive counselling will be trained to provide these treatments at the adolescents’ high schools. Data collection will last from March 2014 to December 2017 and annually thereafter, dependent on the availability of funding.

Participants will receive seven to fourteen 60 min sessions of prolonged exposure treatment (*n* = 45) or supportive counselling (*n* = 45). All assessments will be conducted before treatment, at mid-treatment, immediately after treatment completion, at 3-, 6-, and 12-month follow-up, and annually thereafter. It is hypothesized that PE-A will be superior to SC in reducing PTSD symptoms at post-treatment as measured by the CPSS-I administered by an independent evaluator. It is further hypothesized that PE-A treatment gains will be maintained at 3-, 6- and 12-month follow-ups and annually thereafter.

**Discussion:**

While early indications are that PE-A is an effective treatment for PTSD in adolescents, this study will help determine the effectiveness of PE-A in a South African, community setting (school-based) when task-shifted to nurses, as compared to SC.

**Trial registration:**

Pan African Clinical Trials Registry: PACTR201511001345372, retrospectively registered 11 November 2015.

**Electronic supplementary material:**

The online version of this article (10.1186/s12888-018-1873-x) contains supplementary material, which is available to authorized users.

## Background

Experiences that may be perceived as traumatic, such as experiencing or witnessing either physical abuse, sexual assault, or other forms of violence, are common occurrences among children and adolescents around the world. In school samples in the United States, the prevalence rates of exposure to potentially traumatic events is reportedly between 40 and 70% [1, 2] and in a national study of 2000 children aged 10–16, 24% endorsed being victims of physical assault, sexual assault, or kidnapping [3].

In South Africa, a study conducted in a rural setting found that 67% of children had directly or vicariously experienced a traumatic event [4]. Another, conducted on 10–16-year-old children in a low-income area in Cape Town, found that 95% had witnessed violent events and 56% had experienced violence themselves [5].

In addition to prevalence studies of exposure to traumatic experiences, the literature suggests that adolescents are at an increased risk of developing PTSD with the diagnosis peaking in late adolescence [6].

Two studies conducted in Cape Town, South Africa showed that between 20 and 25% of adolescents met criteria or sub-threshold criteria for PTSD [7, 8] with the most likely pathogenic events identified as sexual assault, physical assault by a family member, and serious accidents. In another South African study of children and adolescents (mean age of 14.3 years) exposed to at least one lifetime potentially traumatic experience, sexual abuse was reported in 53% of participants (42.6% females, 10.6% males), with 64% of violations committed by perpetrators who were known to them [9].

PTSD is often characterized by comorbidity. With the most common co-occurring psychiatric disorder depression [10–13]. The prevalence of depression among adolescents diagnosed with PTSD is reported to be as high as 41% [1, 9]. This is much higher than the rate of depression (8%) amongst their peers [1]. Other common co-occurring diagnoses are separation anxiety disorder, social anxiety disorder, specific phobias, panic disorder, generalized anxiety disorder, and substance abuse disorders [1, 14]. Adolescents who have been assaulted sexually or physically may experience relationship difficulties with peers and caregivers, weaker school performance and behavioral issues [13]. Additionally, those with PTSD may display anger, aggression, self-destructive behaviors, and sexually inappropriate or high-risk behaviors [14], can feel isolated, stigmatized, often struggle with low self-esteem and experience difficulty trusting others [14].

Given the prevalence of PTSD in South Africa and its chronic and debilitating effects in children and adolescents if left untreated, it is a disorder that warrants attention [15, 16]. PTSD can lead to long-term personal suffering that reaches beyond adolescence into adulthood. It can also have serious public health, social and economic implications. In low- and middle-income countries (LMIC’s) there are challenges to both access to and availability of treatment, in particular psychological treatment [8, 17–21] for disorders such as PTSD. This dearth, among other factors, may be attributed to the lack of available skilled treatment providers and concerns among skilled professionals regarding the generalizability of treatments typically developed in higher income, ‘western’ cultural academic settings [19].

Studies have suggested the feasibility of task-shifting the delivery of treatment to community settings and utilizing the services of appropriately trained and supervised non-specialized primary care health workers (NSHWs) [19]. Identifying effective psychological treatments that can be delivered by NSHWs is one of the leading research priorities in the global mental health arena [22]. To demonstrate their portability, evidenced-based trials demonstrating the effectiveness of the task-shifted treatment in LMICs must precede their implementation [20]. The current study aims to provide evidence that both Prolonged Exposure for Adolescents (PE-A) and Supportive Counseling (SC) can be task-shifted to nurses within a community setting for adolescents (i.e., schools).

## Rationale/motivation & originality

Studies conducted to date indicate that trauma-focused cognitive-behavioral therapy (CBT) is an effective treatment for the aftermath of trauma in younger children with sexual abuse trauma [23–29], and other types of trauma [30–32]. Some of these studies were not randomized control trials (RCTs) and most only included younger adolescent participants. It is, therefore, prudent to test the efficacy of trauma-focused CBT in an RCT with adolescents spanning a wider age range (and including older adolescents).

Prolonged Exposure (PE) therapy is a trauma-focused, exposure-based treatment initially developed for adults with PTSD following exposure to diverse types of trauma. There is good empirical support for this therapy, including many RCTs [33]. Due to the relative ease of training, PE has been widely disseminated around the world and following a relatively brief training, non-specialists have been taught to successfully provide PE [34, 35]. PE for adults has been shown to be effective within a community setting [35] and meets the requirements for dissemination. Prolonged Exposure therapy for Adolescents (PE-A), which was based on the adult protocol, also appears to meet these requirements as has been demonstrated in community settings in the USA [36] and in Israel [37]. The comparator treatment, SC, is a non-trauma focused treatment that is widely used in crisis centers and community settings to treat sexually abused children and other traumas [26].

A pilot and feasibility study on psychological treatment of PTSD within a community setting (schools in Cape Town, South Africa) utilizing the services of NSHWs (previously treatment-naive nurses), [38] provided early indications that task-shifting of PE-A and SC for PTSD can be successful.

To our knowledge, there are no other published RCTs for the treatment of PTSD in any population or age group in South Africa. A literature review of all CBT research conducted in South Africa to date, identified 15 studies, with most comprising of a single case design [39].

The proposed research aims to examine the effectiveness of a psychotherapeutic intervention, administered by NSHW’s, to treat the symptoms of PTSD in adolescents exposed to trauma. The study aims are to compare the efficacy of two active treatments, PE-A and SC, in reducing PTSD and other psychopathology symptoms (e.g., depression, anger, other anxiety disorder symptoms, general psychopathology, and global dysfunction), and to examine the relationship between change in PTSD symptoms and change in distress-related cognitions and emotions (e.g., affect regulation, trauma-related cognitions, self-attributions). We also aim to explore whether moderators (e.g., pre-treatment psychopathology, education, parental distress, resilience, self-esteem, perceived social support) and mediators (e.g., treatment satisfaction) are associated with treatment outcome. A further aim of the study is to explore the ability of newly trained counselors (previously treatment naïve), to adequately provide the treatments by adhering to the PE-A and SC treatment manuals. In keeping with international trends to undertake RCTs in naturalistic settings rather than within specialist academic institutions, the present RCT will take place within a community setting, namely, at local schools.

A sub-sample of participants, counselors and teachers who will be involved in the first year of treatment will be contacted during the second year to participate in the qualitative assessment of their subjective experiences by using a nested qualitative component. Triangulation will be employed as it has previously been used to evaluate school mental health program efficacy [40–42] by including semi-structured interviews and focus groups looking at feasibility (barriers/facilitators), acceptability (like/dislike), and impact (pros/cons) as experienced by stakeholders. Although there is literature about the process of seeking mental health treatment as a minor [43] and a quantitative measure to evaluate a youth’s experience with mental health services exists, this will be the first study describing the subjective experiences of adolescents accessing treatment for PTSD.

The study is unique in that it will be the first RCT of CBT in an adolescent population to be conducted within the South African context and that involves task-shifting by identifying counsellors, training and supervising them in a treatment protocol that was originally intended for use and administration by qualified specialized psychologists. It will utilize independent evaluators for the treatment outcome assessments and will examine treatment protocol adherence with independent fidelity ratings.

## Objectives

### Research hypothesis

1. PE-A will be superior to SC as administered by counsellors in reducing PTSD symptoms at post-treatment as measured by the Child PTSD Symptom Scale – Interview (CPSS-I).
2. PE-A treatment gains will be maintained at 3-, 6-, and 12-month follow-up and annually thereafter as measured by the CPSS-I

### Secondary hypothesis

1. At post treatment PE-A will be superior to SC in reducing depression, anger, anxiety, general psychopathology, and global functioning.
2. PE-A treatment gains will be maintained at 3-, 6-, and 12-month follow-up and annually thereafter on measures of depression, anger, anxiety, general psychopathology and global functioning.
3. irrespective of treatment assignment (PE-A or SC), reduction in PTSD symptoms at post-treatment will be significantly and positively correlated with reduction in distress-related cognitions and emotions

### Primary objectives

1. To compare the effectiveness of two treatments administered by counsellors, PE-A and SC, in reducing PTSD symptom severity over 7–14 sessions of treatment.
2. to assess maintenance of PE-A treatment gains on PTSD symptom severity by conducting follow-up assessments at 3-, 6- and 12-month follow-up, and annually thereafter

### Secondary objectives

1. To compare the effectiveness of two active treatments, PE-A and SC, in reducing other psychopathology symptoms (e.g. depression, anger, general psychopathology and global functioning).
2. To assess the maintenance of PE-A treatment gains on secondary outcomes by conducting follow-up assessment at 3-, 6-, 12-month follow-ups and annually thereafter.
3. To examine the relationship between change in PTSD symptoms and change in distress related cognitions and emotions (affect regulation, trauma-related cognitions, and self-attributions).
4. To assess counsellors’ adherence to the PE-A and SC treatment manuals.
5. To evaluate the impact of moderators (e.g. pre-treatment psychopathology, education, resilience, severity of trauma, trauma-related distress, parental distress, social support, self-esteem) and mediators (e.g. treatment satisfaction, compliance, expectation) on outcome.
6. to describe stakeholders (nurses, teachers, adolescents) and their experiences (training, implementation of treatment, access to treatment, barriers, and facilitators to successful implementation) to inform project scalability

## Method

### Design

The study design is reported in line with the SPIRIT 2013 Statement (Explanation and elaboration: Guidance for protocols of clinical trials) [44].

**Fig. 1:**
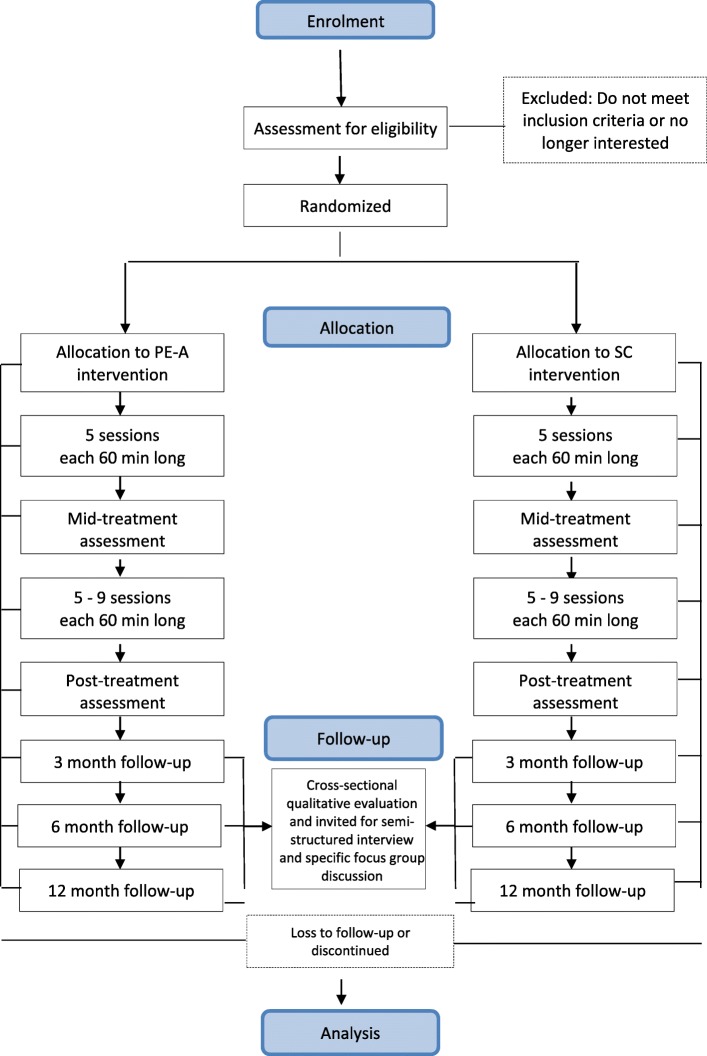
Study Consort Diagram, Consort diagram

Figure 1 provides an overview of the study design.

A single-blind permuted block randomized control design will be used. The proposed research aims to examine the effectiveness of a psychotherapeutic intervention administered by counsellors to treat the symptoms of PTSD in adolescents exposed to trauma. The aim is to conduct a randomized controlled trial (RCT) to compare PE-A to SC to determine the comparative effectiveness in reducing PTSD and associated symptoms in adolescents.

The study will compare the efficacy of PE-A to SC. Participation will be voluntary. All participants, who meet full PTSD or sub-threshold PTSD criteria, will be scheduled for a pre-treatment assessment with Independent Evaluators (IE). The one IE will be an experienced diagnostician and clinical psychologist and the other an experienced psychiatric nurse. Adolescents will be randomly assigned on a 1:1 ratio to receive either PE-A or SC.

Treatment will consist of 7–14 sessions of either PE or SC. The number of sessions each participant receives will be based on the weekly self-report measure of PTSD symptoms (Child PTSD Symptom Scale – Self Report; CPSS-SR); however, this will range between a minimum of 7 sessions to a maximum of 14 sessions. A 70% symptom reduction is chosen as sufficient for treatment termination.

After session five, all participants will receive a mid-treatment evaluation by the IE. The IE will again assess the participants upon termination of treatment. Participants will then enter the non-treatment follow-up phase. Participants who prematurely terminate therapy (before 70% symptom reduction or 7 sessions) will undergo a post-last session assessment with the IE and will then enter the follow-up phase. Follow-up assessments will be conducted at 3-, 6-, and 12 months post-treatment and annually thereafter. The IE assessments will comprise both structured clinical interviews and self-report measures. The assessments will cover PTSD severity, related psychopathology, general psychopathology, and PTSD-related cognitions. At the 12-month follow-up, participants who receive SC and are deemed to still have clinically significant PTSD symptoms (as determined by the IE at 12-month follow-up assessment), will be offered PE-A. To add to the rigor of the study and demonstrate the long-term efficacy of the intervention, there will be annual posttreatment follow ups, dependent on the availability of funds for this purpose.

A cross-sectional qualitative evaluation will take place during the follow up assessments of the adolescents who have completed their treatment. Adolescents who participated in the study in the preceding year (regardless of completion or outcome) will be invited to participate in semi-structured interviews with an independent psychologist. Similarly, there will also be separate focus groups for the nurse counsellors and the contact persons at the schools who had been part of the study during the preceding year.

### Ethics approval

This study has been approved by the Health Research Ethics Committee at Stellenbosch University (N12/06/031) and will be conducted according to the ethical guidelines and principles of the international Declaration of Helsinki, South African Guidelines for Good Clinical Practice and the Medical Research Council (MRC) Ethical Guidelines for Research.

Permission was also obtained from the Western Cape Education Department (WCED) to offer treatment at the participants’ schools. Permission from the WCED was conditional on not disclosing the identities of the participating schools.

Consent will be obtained from parents of potential participants in the study. Informed consent and assent will be obtained by the IE when determining eligibility and prior to randomization to treatment. A participant may leave the study at any time. Study procedures as well as potential risks, benefits and treatment alternatives will be explained at the time participation in the study is discussed. Participants will be clearly informed of the voluntary nature of their participation and their right to terminate without any penalty.

The one IE, an experienced Clinical Psychologist, will screen participants for other psychopathology and will not include a potential participant if the principal diagnosis is not PTSD in response to a traumatic event.

Participating schools’ headmasters and educators will be informed that the study entails the treatment of adolescents that have experienced a trauma. In this way adolescents will not be identified in terms of the nature of their trauma. Counsellors will keep all study and personal information about participants confidential.

Participants will receive all written material in their language of choice (Afrikaans, English and Xhosa).

All counsellors will receive weekly supervision, on every participant under their care. Counsellors will have unrestricted access to the supervisor. Participants in both PE-A and SC will be informed that they can contact their counsellor between sessions should they experience a crisis or are distressed enough to need support. If a counsellor is concerned about a participant’s psychological welfare, the supervisor will arrange for a psychological assessment. Although unlikely, should significant negative emotional reactions develop that require other forms of intervention, the participant will be withdrawn from the study and will be appropriately referred.

Should the parent be unable to provide informed consent in person for the qualitative addition to the study, the following procedure will be used:

1. Parent contacted by telephone, given information, and read the consent form. Parent will be given opportunity to ask questions. Consent via the telephone will be recorded.
2. Teenager will be picked up for interview. Before the interview begins, the child will provide their written assent.
3. When the teenager leaves the interview, he/she will take the informed consent form/information letter home for the parent to sign. A witness will co-sign the consent form.
4. to be picked up for the focus group, the child must show the driver a signed copy of the consent form. The consent form will be collected at the focus group and a copy will be sent back with the teenager for the parent’s records

### Participants

Adolescents aged 13 to 18 years, who have experienced an interpersonal trauma, will be included in the study. Adolescents must present with chronic (at least 3-months) full PTSD or sub-threshold PTSD to be included. Sub-threshold PTSD is defined as having at least 1 re-experiencing symptom, at least 2 avoidance symptoms, 2 arousal symptoms and a score of 11 on the CPSS. Providing that PTSD is deemed to be the primary disorder requiring treatment, adolescents with comorbid mood disorders, anxiety disorders, substance use disorders and ADHD will also be included. Adolescents with conduct disorder, a primary substance abuse problem and psychotic disorders will be excluded.

For the qualitative study, stakeholders should have been involved in the study during the previous year (to make sure that they still remember their experiences). Stakeholders will be included even if; a) the adolescent did not complete the treatment intervention; b) the nurse did not complete the yearlong commitment; c) the nurse did not gain experience in implementing both treatment strategies; or, d) the school did not yield any recruits.

### Recruitment

Participants for the RCT will be adolescents recruited from lower socio-economic status community schools around Cape Town. A recruiter will address the children during assembly and in individual classes. The recruiter will explain the definition of a traumatic event, will describe typical events that would be considered a qualifying traumatic experience, will explain the diagnosis of PTSD and explain that this study aims to compare the effectiveness of two treatments when administered by nurses within the community (school). Adolescents who think that they could benefit from treatment will complete a PTSD screening instrument in private. Prospective participants who indicate that they have experienced a trauma and whose scores are beyond the cut-off for inclusion will be scheduled for a pretreatment assessment. For those who meet the criteria, a pre-assessment interview will be scheduled with the adolescent and their parent or guardian.

In the nested qualitative study, adolescents completing treatment in the preceding year will be invited to participate in a treatment specific focus group with similar adolescents and will participate in individual interviews. The topic guide and other standard operating procedures may be seen in (Additional file 1: Table S4 – S4c). Nurses/counsellors who had been trained in the previous year will be invited to attend a focus group (Additional file 1: Table S5 – S5c) and similarly, contact persons at school (teachers) will be invited to attend a focus group for representatives from host organizations (Additional file 1: Table S6 – S6b). All stakeholders who will have been involved in that time frame will be contacted by phone to set up an information/informed consent session for the qualitative portion of this study.

### Procedure

Counsellors will be sourced from a one-year advanced diploma in psychiatric nursing student group at the University of Stellenbosch. The definition of a counsellor for the purpose of this study will be a qualified nursing sister. Both treatments (PE-A and SC) will be provided by each of these counsellors to prevent confound of therapist effects. The nursing sisters will only be reimbursed for petrol/transport costs. Counsellors will be identified and trained every year. This will result in counsellors not seeing more than 4 participants each, thus remaining relatively inexperienced over the period of the study.

The training will be 4 days in duration, 3 days devoted to training in the PE-A protocol and the 4th-day to training in SC. The SC training will be followed by an additional 16 h of practical training. The training will culminate in the ability to implement the PE-A and SC skills following the two treatment manuals, respectively. Every counsellor will have a file with all the information needed for each participant they are treating. Counsellors will, therefore, be able to follow the treatment steps and have all the information required at the ready for every participant. Weekly supervision will occur throughout the duration of the study.

Identified individual participants enter a pre-treatment preparation phase conducted by an IE. During this time the IE will explain the study (nature of treatments, random assignment, duration of treatment, measures and follow-up procedures), obtain informed consent, and address logistical issues.

Participants will be randomly assigned to a counsellor and to a treatment condition. The randomization schedule will be created by an independent statistician and only provided to the project coordinator. The project coordinator, the only one with access to the randomization scheme, will contact the counsellor. The counsellor will be informed of the treatment condition that the client has been randomly assigned to. The counsellor will contact the school and the adolescent and set up the first appointment. Both treatments will be administered by the counsellors within the high-school setting.

Following the pre-treatment preparation, both PE-A and SC will consist of 7–14 sessions, lasting 60 min each.

The CPSS-SR will be administered at the beginning of each session in order to monitor treatment progress. From session 7, the percentage improvement from the pre-treatment baseline will be measured. When improvement reaches 70% or better, the counsellor will share the outcome with the participant and start the termination process.

The follow-up assessments will be administered by an IE blind to the treatment condition.

#### Monitoring treatment integrity

All treatment sessions will be video recorded. Ten percent of treatment sessions will be randomly selected for protocol adherence ratings. Oversampling will take place on the sessions in which the rationale of SC or PE-A is explained to track adherence to the protocol during these critical sessions. Counsellors will make note of any mention of the trauma during the SC sessions.

#### Qualitative portion of study

Adolescents who agree to participate in the study will be interviewed by an independent evaluator and will participate in a treatment specific focus group. These interviews will be audio-recorded.

The focus groups with the nurse counsellors and school contact personnel will be conducted at Stellenbosch University by an independent clinical psychologist. A co-facilitator will record field notes and manage the audio-recording.

### The PE-A treatment group

The PE–A program consists of 7 to 14 weekly, 60 min sessions. Treatment comprises 8 modules. Homework exercises provide the opportunity for practice outside of the session. Module 1 includes presentation of the treatment rationale. Module 2 includes information gathering, identifying an index trauma, and conducting a breathing retraining exercise. Module 3 presents common reactions to trauma. Module 4 includes discussion of the rationale for in vivo exposure (confronting trauma reminders in real life), construction of the in vivo hierarchy, and assignment of in vivo homework. Module 5 includes presentation of the rationale for imaginal exposure (revisiting and recounting the traumatic memory), conducting imaginal exposure for 15 to 45 min, and processing this revisiting experience. This module is repeated for 2 to 5 sessions. In module 6, the imaginal exposure focuses on the worst moments of the trauma. Module 6 is repeated for 4 to 7 sessions. Module 7 focuses on generalization of skills learned in treatment and on relapse prevention. Module 8 comprises a “final project,” such as making a collage detailing the trauma and the gains made in treatment.

Table 1 provides a detailed outline of the PE-A treatment.

**Table 1:** Outline of PE-A treatment, A detailed outline of the PE-A treatment

Major focus	Goals	Sessions (ideal)	Present at session
Phase 1: Psychoeducation and treatment planning			
Module 1: Treatment Rationale Tool 1: Breathing Retraining	1. Build rapport 2. Give overview of treatment 3. Introduce treatment rationale to the adolescent and the parent or guardian 4. Provide the adolescent with a tool to control anxiety	1,2	Adolescent, Guardian (Full or partial)
Module 2: Gathering Information	Gather information as the adolescent tells about the traumatic event.	1,2	Adolescent
Module 3: Common Reactions	1. Describe common reactions 2. Discuss the adolescent’s own reactions to trauma	2, 3	Adolescent, Guardian (partial)
Phase 2: Exposure			
Module 4: Rationale for Exposure Tool 2: Real Life Experiments Tool 3: Stress Thermometer	1. Review rationale for real-life experiments/In –vivo Exposure 2. Discuss how confronting feared situations reduces anxiety reactions	3,4	Adolescent, Guardian (partial)
Module 5: Dealing with avoidance. Tool 4: Construction of hierarchy for real life experiments	1. Create in vivo hierarchy 2. Choose an initial target for in vivo exposure	3,4	Adolescent, Guardian (partial)
Module 6: Recounting the trauma Tool 5: Imaginal Exposure	1. Explain why it is helpful to recount the memory of the trauma 2. Reliving the memory through writing or talking	4,5,6,7	Adolescent
Module 7: Recounting the Trauma Tool 6: Worst Moments	Process the most difficult parts of the memory	7,8,9,10, 11,12,13	Adolescent
Phase 3: Relapse Prevention and Graduation			
Module 8: Generalization and Relapse Prevention	1. Review progress 2. Discuss the efficacy of various tools acquired in therapy. 3. Introduce generalization training and relapse prevention 4. Discuss the outcome of the therapy	10,11,12,13, or 14	Adolescent, Guardian (partial)
Module 9: Graduation	1. Review Progress with Parents/Guardians 2. Celebrate accomplishments 3. Reiterate Relapse Prevention Strategies	10,11,12,13, or 14	Adolescent, Guardian (partial)
Modules 1–9: Family intervention	1. Foster relationship with guardian(s) 2. Inform the Guardian about the progress of treatment. 3. Address obstacles to treatment.	Family interventions are included in each module. Up to 3 additional sessions as necessary to meet goals of the module. To include parents with or without adolescent.	

### The SC treatment group

SC consists of 7 to 14 weekly, 60 min sessions of client-centered therapy. SC is based on the Traumagenic Dynamics Model of symptom formation after child sexual abuse and the Rogerian psychotherapy model. Supportive counselling sessions focus on establishing a trusting, empowering, and validating therapeutic relationship. Participants are allowed to choose when, how, and whether or not to address their trauma. In session 1, participants are oriented to SC.

Counsellors provide active listening, empathy, and encouragement to talk about feelings and express beliefs in the participant’s ability to cope. Participants direct the sessions. Counsellors note discussion of the trauma and the time devoted to such discussions. Tools such as problem solving and keeping a diary will be taught to participants.

Table 2 provides a detailed outline of the SC treatment program.

**Table 2:** Outline of SC treatment, A detailed outline of the SC treatment

Major Focus	Goals	Sessions (ideal)	Present at session
Phase 1: Psychoeducation and treatment planning			
Module 1: Information gathering and Treatment Rationale	1. Build rapport 2. Gather information about the trauma 3. Give overview of treatment	1,2	Adolescent, Guardian (Full or partial)
Module 2: Treatment Planning Tool 1: Daily Diary	1. Gathering information about everyday problems 2. Give the rationale for supportive counseling 3. Introduce Daily Diary	2,3	Adolescent, Guardian (partial)
Phase 2: Treatment			
Module 3: Crisis Intervention and Coping Tool 2: Positive Problem Solving	1. Review Daily Diary 2. Provide Support 3. Practice Problem Solving	3–9 or 13	Adolescent
Phase 3: Relapse Prevention and Graduation			
Module 4: Generalization and Relapse Prevention	1. Review progress 2. Discuss the efficacy of various tools acquired in therapy. 3. Introduce generalization training and relapse prevention 4. Discuss the outcome of the therapy	10.11.12.13,or 14	Adolescent Parent (partial)
Module 5: Graduation	1. Review Progress with Parents/Guardians 2. Celebrate accomplishments 3. Reiterate Relapse Prevention Strategies	10,11,12,13, or 14	Adolescent, Guardian (partial)
Modules 1–5 Family intervention	1. Foster relationship with parent (s) 2. Inform the parents about the progress of treatment. 3. Address obstacles to treatment.	Throughout the treatment. Up to 3 additional sessions as needed to include parents with or without adolescent.	

### Measures

A pilot study of 12 participants [38] was used to determine the final assessment battery (from the included battery) that will be used for the study. The pilot study was also used to identify any logistical or treatment protocol implementation difficulties.

The assessment battery that will be administered to all adolescents who are eligible on screening is detailed below in Table 3.

**Table 3:** Measures used in assessment, Measures used and indication when used in assessment

Domain	Measure	Assessing	Informant	Time (min)	Pre T1	Mid T2	Post T3	3, 6, 12 month FUs
Diagnostic Information	MINI-KID [45]	PTSD and other symptoms	*IE*	90	X			
	MRC Demographic Questionnaire	Demographic Information	*Parent*	10				
Psychopathology	CPSS [46, 47]	PTSD severity	*IE*	10	X	X	X	X
	CGAS [48]	*General Functioning*	*IE*	*5*	X	*X*	*X*	*X*
	BDI [49, 50]	*Depression*	*Adolescent*	*10*	X	*X*	*X*	*X*
	STAXI [51, 52]	*Anger*	*Adolescent*	*10*	X	*X*	*X*	*X*
	CBCL-YSR [53]	*General psychopathology*	*Adolescent*	*20*	X		*X*	*X*
	MASC [54, 55]	*Anxiety*	*Adolescent*	*5*	X	*X*	*X*	*X*
	PESQ [56]	*Substance use*	*Adolescent*	*15*	X	*X*	*X*	*X*
Cognition & Emotion	C-PTAS [57]	Trauma-related cognitions	*Adolescent*	10	X	X	X	X
	CAPS [58]	Attributions and perceptions	*Adolescent*	10	X	X	X	X
	PTCI [66]	Trauma-related cognitions	*Parent*	10	X		X	X
	NMR [59, 60]	Affect Regulation	*Adolescent*	10	X		X	X
	MSPSS [61, 62]	Social support	*Adolescent*	10	X	X	X	X
	Rosenberg [63]	Self-esteem	*Adolescent*	3	X	X	X	X
	CD-RISC [64]	Resilience	*Adolescent*	5	X	X	X	X
Symptoms – Parents	PDS [65]	PTSD	*Parent*	20	X		X	X
	BDI [49, 50]	Depression	*Parent*	10	X		X	X
Treatment variables	Expectancy-A	Tx expectancy	*Adolescent*	2	X			
	Satisfaction-A	Tx Satisfaction	*Adolescent*	2		X	X	X
	CPSS [41]	PTSD severity	*Adolescent*	10	Every session			
	Treatment compliance	Compliance with the treatment	*Therapist*	2		X	X	X

All measures will be obtained by an IE at baseline, mid-treatment (after 5 sessions), and post-treatment, and again at 3-month, 6-month and 12-month follow-up, and in following years at annual intervals, dependent on the availability of funds.

#### Diagnostic information

##### *The Medical Research Council Demographic Questionnaire* (MRCDQ)

The MRCDQ will be used to obtain each adolescent’s age, grade, gender and alcohol and drug use history from the adolescent, educator or parent. Information on the parents will include, age, educational level, marital status, income, employment, and their alcohol and drug use history.

##### *The Mini International Neuropsychiatric Interview for Children and Adolescents (MINI-KID;* [45]*)*

The MINI-KID is a semi-structured interview, administered by a trained clinical interviewer, covering current and lifetime disorders. It has excellent test-retest reliability (0.64–1.00).

#### Psychopathology measures

##### *The Child PTSD Symptom Scale–Interview (CPSS-I*; [46]*)*

The CPSS-I will be used in conjunction with the MINI-KID to assess PTSD diagnosis and PTSD symptom severity. The CPSS interview (8–18 years), is based on DSM-IV criteria and provide a Total-, re-experiencing-, avoidance- and hyperarousal score. The CPSS-I has 17 items that correspond with the DSM-IV and an additional 7 items. The CPSS-I Total score ranges from 0 to 51 and each item is rated from 0 to 3. Internal consistency ranged from .70–.89 for the total and symptom cluster scores. Test-retest reliability was good to excellent (.84 for the total severity score, .85 for re-experiencing, .63 for avoidance and .76 for arousal). Convergent validity was high (.80) between the CPSS and the Child Posttraumatic Stress Reaction Index [47]. A cutoff Total score of 11 or higher on the CPSS yielded 95% sensitivity and 96% specificity of diagnosis. The CPSS-I Total Score will be used as the primary outcome measure.

##### *The Child PTSD Symptom Scale – Self Report (CPSS-SR*; [46]*)*

The CPSS-SR is a self-report version with psychometric properties that are equivalent to the CPSS-I and will be utilized in every session as a repeated measure of PTSD symptoms and will also be part of the pre-treatment assessment package. It will be administered weekly as a self-report to monitor treatment progress and will be used to decide when to terminate treatment.

##### *The Children’s Global Assessment Scale (CGAS*; [48]*)*

The CGAS will be used by the IE to provide a measurement of general functional impairment and as a secondary outcome measure by the IE. It is an appropriate tool for children from age 4–18. The CGAS has demonstrated good inter-rater reliability (*r* = .85) when administered by trained clinicians.

##### *The Beck Depression Inventory (BDI;* [49, 50]*)*

The BDI, which has been normed for adolescents, will be used to assess depression. The BDI split half-reliability is .93, and correlations with clinician ratings of depression range from .62 to .65 [49]. The BDI will be used by the IE as a secondary outcome measure.

##### *The Spielberger State-Trait Anger Expression Inventory-2 (STAXI-2;* [51, 52]*)*: 1999)

The STAXI-2 is a 57 item self-report measure. It has a State-Anger subscale measuring the intensity of angry feelings and the extent to which a person feels like expressing anger at a particular time and the STAXI Trait-Anger scale that measures how often angry feelings are experienced over time, using a 4-point Likert scale. Anger control is measured by presenting descriptions of responses and participants rate how often they behave in the manner described when angry, also using a 4-point Likert scale. Norms are available for adolescents 16–19 years of age. Internal consistency (.88) is good. High scores on the STAXI-2 scales (75th percentile) indicate anger experience and expression that interferes with normal functioning. The STAXI-2 will be incorporated as a secondary measure.

##### *The Child Behavior Checklist-Youth Self Report (CBCL-YSR*; [53]*)*

A 112-item self-report screening tool for behavioural and emotional problems in children and adolescents. In this study, we report the Externalizing, Internalizing and Total Problem scores. Raw scores are converted to T-scores. On all three scales, a T-score of 70 and above is in the ‘Clinical’ range. A T-score of 65–69 is in the ‘Borderline Clinical’ range. It is normed for ages 11–18. The Total Problem Score has been shown to have excellent test-retest reliability (.87). The Total Problem Score, Internalising behaviour and Externalising behaviour scales will be used by the IE as secondary measures.

##### *The Multidimensional Anxiety Scale for Children (MASC*; [54, 55]*)*

The MASC is specifically designed to assess a wide spectrum of common anxiety symptoms in youth. The items are distributed across 4 major factors; physical symptoms, social anxiety, harm avoidance and separation anxiety the ratings are done on a 4-point Likert rating scale. The scale has been shown to be invariant across gender and age and shows excellent internal consistency and test-retest reliability [53]. A study showed that the same 4-factor structure applies to adolescents in the Cape Town metropole, when compared to North American and Icelandic samples [55]. Another important finding was that the 4-factor structure applied equally well to males and females, making between group comparisons possible within this context. The MASC also measures separate dimensions of anxiety amongst various racial groups, which makes it suited to discriminate patterns of anxiety in subgroups of youth with anxiety disorders.

##### *The Personal Experience Screening Questionnaire (PESQ*; [56]*)*

This measure was developed as a screening tool for drug use in adolescents. This scale provides a global measure of problem severity, by indicating to what extent adolescents are psychologically and behaviourally involved with drugs. The score on the problem severity scale varies from 0 to 54, with a score of 30 indicating a problematic level of drug use. Internal consistency ranges from .90 to .95 across normal, delinquent and substance-abusing adolescents. The PESQ Problem Severity score will be used as a secondary measure by the IE.

#### Cognition and emotion measures

Three measures of dysfunctional cognitions related to trauma will be administered to investigate the role of cognition in mechanisms of change:

##### *The Child Post-Trauma Attitudes Scale (C-PTAS*; [57]

This is a 30-item self-report measure for children 8 and older and generates a total score and 3 subscale scores for negative post-traumatic cognitions. The subscales measure perceptions about the dangerousness of the world, a sense of own competence, and isolation. The measure also yields a total score which ranges from 0 to 120. Internal consistency was moderate to high, with alphas ranging from .66 to .82 for the total C-PTCAS score and three subscales. The C-PTAS will be used as a secondary measure by the IE.

##### *The Children’s Attributions and Perceptions Scale (CAPS;* [58]

The CAPS is an 18-item scale that has been normed as an interview but will be used as a self-report measure. The CAPS rated on a 5-point Likert Scale has four subscales: Feeling Different from Peers, Personal Attributions for Negative Events, Perceived Credibility, and Interpersonal Trust. Internal consistency for the subscales ranges from .63 to .73. Test-retest reliability ranges from .60 to .82 for the subscales and is .75 for the total scale.

##### *The Negative Mood Regulation questionnaire (NMR;* [59]*)*

This is a 30-item measure of beliefs that individuals have, which, when they are in a bad mood, can effect a change to help them feel better. Higher scores indicate better expectancies around the ability to control negative mood. Test-retest reliability for the NMR was acceptable (.67–.78). Although this measure was not designed specifically to be used in adolescents, it has been used successfully in studies of depressed suicidal and non-suicidal adolescents [60]. The NMR will be used as a secondary measure by the IE.

Additionally, the following three adolescent self-report measures will be administered:

##### *The Multidimensional Scale of Perceived Social Support (MSPSS*; [61, 62]

The MSPSS is a 12-item self-report inventory that measures the perceived adequacy of social support from 3 sources; family, friends and significant other. The ratings are made on a 7-point Likert-Scale. The psychometric qualities of the MSPSS were evaluated within a South African context with the internal reliability of the scale and subscales found to be high (Cronbach alpha coefficients > 0.85) [62]. The MSPSSis psychometrically sound and has excellent internal validity in South African youth.

##### *The Rosenberg Self-Esteem Scale* [63]

This is a 10-item self-rating scale to assess self-esteem. The ratings are done on a 4-point Likert scale. The scale has been evaluated across multiple cultural contexts [63], including Western, Eastern and African contexts.

##### *The Connor-Davidson Resilience Scale (CD-RISC*; [64]

The CD-RISC is a 25-item self-report measure to assess resilience. Ratings are done on a 5-point Likert Scale.

#### Parental symptoms - moderators

Parental distress will be assessed as moderators of outcome. If parents themselves suffer from PTSD following a trauma, they might interfere with the exposure procedures.

##### *The Posttraumatic Diagnostic Scale (PDS*; [65]

The PDS will be used to measure the total and subscale severity scores and categorical classification of PTSD.

##### *The Beck Depression Inventory (BDI;* [49, 50]

The BDI will assess depression as it can be widely used in a variety of populations, including trauma victims [67] and the.

##### *The Post-Traumatic Cognitions Inventory (PTCI*; [66]

The PTCI is a 36-item self-report instrument that assesses dysfunctional post-trauma cognitions. Items are rated on a 7-point Likert Scale; this will be completed by the parent/guardian.

### Treatment related variables - mediators

Three self-report measures will be administered to examine their mediating effects on treatment outcome:

- *Expected Treatment Outcome (ETO)* will be provided by the adolescent on a 5-point Likert scale from “I expect this treatment to help me a lot” (5) to “I don’t expect this treatment to make any difference in my condition” (1).
- *Treatment satisfaction (WIAC)* will be provided by the adolescent on a 5-point Likert scale from “I am extremely satisfied with this treatment, it helped me a great deal” (5) to “I am not at all satisfied with this treatment, it did not help my condition” (1).
- *Treatment Compliance* will be assessed by the counsellor by ranking the adolescent’s compliance with treatment and homework on a 5-point Likert scale.

#### Qualitative study

Topic guides and interview schedules for the different qualitative groups are included in (Additional file 1: Table S4 – Table S6c). The Standard Operating Procedures (SOP) for adolescents, the initial telephone contact procedure with the adolescent’s parent, and the interview schedule and focus group topic guides to be used in the qualitative interviews can be found in (Additional file 1: Table S4, S4a and S4b). The SOP outline for the nurse counsellors, the initial telephone contact, the focus group topic guide and a questionnaire to be completed can be found in (Additional file 1: Table S5, S5a, S5b and S5c). The details of the SOP for teachers, the initial telephone contact and the focus group topic guide can be found in (Additional file 1: Table S6, S6a and S6b). The SOP for data coding and thematic analysis are presented in (Additional file 1: Table S7) and the details of the SOP for transport arrangements of participants to the Stellenbosch University Medical Campus are presented in (Additional file 1: Table S8).

### Data management and monitoring

Acquired data will be entered immediately into a database constructed using SPSS. Each entry form will be restricted to the possible range of items. Data entry and verification will be conducted independently by study assistants.

### Statistical analysis

#### Preliminary data analysis

Demographic characteristics and outcome measures at pre-treatment assessment will be examined using t-tests (for continuous variables) or chi-square tests (for nominal variables). These comparisons will enable the identification of control variables for use in later analysis for comparing treatment differences. The focus will be on both statistical and clinical significance of group differences (means and proportions of variants explained).

#### General considerations for data analysis

Descriptive statistics will be gathered in order to determine if variables have a normal distribution and variables that violate assumptions of normality will be transformed. If this does not improve the normality of distribution, distribution free statistics will be used.

The study has pre-, mid-, post-treatment, three follow-up assessments and annual follow-up thereafter. This creates clustered data due to repeated measures. To examine treatment difference, generalized estimating equations (GEE) and a linear mixed-effects model approach (LMM) will be employed. Both approaches accommodate missing data. LMM will be the main analytical tool, particularly for estimating between-participant variability; GEE will be used to assess the robustness of the population parameters, such as treatment effects.

Analyses correlating the change in PTSD symptoms with each of the distress-related cognition and emotion variables at post-treatment will be performed, by pooling data across treatment conditions. Product-moment and Spearman’s rho correlation coefficients will be used.

Correlation analyses correlating the change in PTSD symptoms with each of the distress- related cognition and emotion variables at post-treatment, by pooling data across treatment conditions, will be performed. Product-moment and Spearman’s rho correlation coefficients will be used.

### Exploratory analyses

Moderators such as (pre-treatment psychopathology, education, resilience, self-esteem, perceived social support, type of trauma (sexual assault vs. physical assault), treatment expectancy and mediators (treatment satisfaction, treatment compliance, and amount of time spent with guardians in treatment sessions) will be explored to determine if they are related to treatment outcome.

In each analysis a time-by-treatment-by-moderator interaction for each of the moderators will be investigated. For each mediator, structural equations models (SEM) to model the mediated relationship to treatment conditions and each primary outcome of interest will be performed. With three assessment points at pre-, mid- and post-treatment, there is only one direct and one indirect effect of treatment condition on the response at post-treatment [67–69].

#### Thematic analysis procedure for qualitative aspect

All audio-recordings from interviews and groups will be independently transcribed twice, once by the facilitator and once by a research assistant. Anticipated themes will be informed by the topic guides/interview schedules (feasibility, acceptability, impact, barriers, facilitators). Thematic content analysis will be done using Atlas.ti software (Additional file 1: Table S7).

### Sample size

It is believed that the within-group effects for both PE-A and SC in this study will be comparable to the effect sizes in previous studies examining these treatments in children and adolescents and adults [26, 36, 68, 69]. Using these data, it, is suggested that a total of 64 completers is necessary to provide 80% power for a two-tailed alpha level of 0.1. The literature suggests that as many as 30% of PTSD study entrants will terminate prematurely, i.e., drop out. A total of 90 entrants will thus result in 64 completers.

## Discussion

This is the first RCT using evidence-based CBT for PTSD in traumatized adolescents within a South African context. In addition, the study will be assessing the effectiveness of task shifting in a limited resource environment, using counsellors with minimal to no previous experience in psychosocial treatment of PTSD, which will be vitally important from a public mental health perspective. Should the results of this study demonstrate that PE-A is an effective psychotherapeutic intervention for treating symptoms of PTSD in adolescents exposed to trauma, when administered by trained counsellors, the implications for treatment recommendations may be wide-ranging. Thus, community-based personnel could be trained relatively easily to deliver the treatments within the sufferers’ environment, which in turn, would make effective treatments more readily accessible as well as more cost-effective.

### Potential risks

(i) Participants can develop mild to moderate emotional discomfort or frustration associated with interviewing, or completing lengthy questionnaires; (ii) participants may experience subjective distress during treatment; (iii) all participants are exposed to the risk of relapse during the non-treatment follow-up; and, d) participants might suffer from stigma at the schools they attend.

There is the risk that some participants accidentally state identifiable information on the recording device that is recognizable by the research assistant-transcriber.

During focus groups, (i) participants may experience mild emotional discomfort in situations where there are differences of opinion from theirs or (ii) when talking about aspects of treatment. (iii) Adolescents may be disappointed that they did not have the same therapeutic experience as another participant or (iv) they may fear being recognized by a fellow participant.

The risks for nurses is that they may experience anxiety concerning negative consequences in relation to voicing dissatisfaction about training and/or supervision conducted by the PI.

### Protection against risks

Participants not deemed study eligible (see exclusion criteria) or who themselves do not assent or their parents or guardians do not consent to participation in the study will be offered clinically appropriate treatment referrals outside the study.

Prior to exposure-based interventions, informed consent/assent will be obtained from eligible adolescent participants to reduce possible risks of mild to moderate anxiety when exposed to anxiety-provoking images and situations in treatment sessions, at home or doing homework. Each participant will be given a rationale for PE and advised of the procedure and the temporary effects that the procedure might have. Participants will be required to give assent prior to every exposure session or task. Exposure therapy has been shown to provide benefits. While some individuals fail to benefit from an intervention, very few report adverse effects. The exposure is individually titrated; in-vivo exposure starts with situations that elicit moderate anxiety and progress to more anxiety-provoking situations and triggers.

Risks associated with SC may include non-reduction of PTSD symptoms. All participants in this treatment condition who retain a PTSD diagnosis or manifest significant trauma-related symptoms, will be offered PE-A at the end of treatment.

In the nested qualitative study, to manage potential emotional risks, the focus groups and interviews will be conducted by a clinical psychologist. Participants will be encouraged to use code names while the recording is on and the recording will be identified by a code. Only the two transcribers will have access to the audio-recordings and transcribe using earphones in a private location. Transcribers will sign a confidentiality agreement. All identifiable information will be removed from the transcription prior to analysis. The PI will not be present during the focus group discussions and all feedback to him will be de-identified.

Adolescents will attend intervention specific (PE-A or SC) focus groups to protect them from exposure to an unfamiliar treatment experience. If there are enough interested adolescents, the groups can be further separated based on gender. Rules regarding confidentiality and respect will be reinforced throughout focus groups.

Participants’ reactions to study assessments and treatment will be closely monitored, and negative reactions will be addressed therapeutically. To avoid or reduce mild to moderate emotional discomfort or frustration associated with psychiatric interviewing or filling out questionnaires, adolescents and their parents will be allowed breaks as needed during in the assessment phases. Additionally, the intake assessment can be spaced over visits, if needed.

Participants in both conditions will be informed by their respective counsellors that they may call between sessions should they experience a crisis or are distressed enough to need psychological support. If a counsellor becomes concerned about a participant’s psychological welfare, contact will be made with the supervisor and PI who will arrange for psychological evaluation. Although unlikely, if substantial negative emotional reactions develop that require additional interventions, participants will be withdrawn from the study and referred to public health treatment providers to receive these services. Such identification can be made by IEs or study counselors, in consultation with the PI.

In cases where danger to self or others is identified, the adolescent will be withdrawn from the study and clinically appropriate emergency service procedures will be implemented. If previously undocumented sexual or physical abuse is discovered during consent or treatment, we will implement standard procedures for notifying the appropriate social services and provide referrals to local public mental health treatment centers. If a social work referral is required during treatment, the adolescent may or may not be continued in the study depending on what is in the best interest of the participant. This decision will be made by the PI and the treatment team in consultation with the adolescents’ primary caregiver, provided that this caregiver is not the perpetrator.

All study data will be confidential. The following steps will be taken to minimize risks to confidentiality: a) evaluation protocols and treatment record forms will be kept in locked cabinets in the IE office; b) participant identity will be disguised using ID numbers keyed to a master list; c) all recordings will be transferred to removable storage that will be kept in locked storage in locked rooms; d) data will be entered directly onto computer files that will be password protected; and e) computer files will carry only participant numbers for case identification. All project staff will be trained in the importance of confidentiality. If the results of the study are published, data that might reveal the identity of a participant will be disguised.

### Data safety and monitoring review board

In view of the potentially high risks associated with counsellors administering psychotherapeutic interventions in a vulnerable population (traumatized adolescents with PTSD), a Data Safety and Monitoring Board (DSMB) will be established for this trial. The DSMB will include a child psychiatrist, clinical psychologist, statistician and an ethicist. The DSMB will meet at regular intervals upon completion of the pilot study, as well as every 6 months during the study, to review cumulative data and to evaluate the safety of human participants, study conduct, and the scientific validity and integrity of the data. The DSMB will report back on its findings to the study team and to the Health Research Ethics Committee (Faculty of Health Sciences, Stellenbosch University) via the Principal Investigator. Audio-recordings will be stored in a locked cabinet in the researcher’s office and destroyed after 5 years.

### Study implications

Information from this study can directly inform and benefit both policy makers and participants by guiding appropriate treatment planning and providing some therapeutic answers to a population in need. Thus, if the hypotheses of this study are supported by the data, dissemination of these treatments, to be delivered by trained and supervised previously psychotherapy-naïve health care personnel in general practice would be recommended. Participants in the treatment study will benefit from receiving well-delivered treatment for PTSD and the task-shifted counsellors will benefit from learning evidence-based effective treatments for PTSD under close and supportive supervision. Participants may also benefit from knowing that they have contributed to the scientific understanding of PTSD and its treatment that may help other adolescents struggling with similar problems and their community care providers.

## Additional file

Additional file 1: Topic guides and interview schedules for the different qualitative groups, Written standard operating procedures. (DOCX 84 kb)

## Abbreviations

BDI: Beck Depression Inventory
CAPS: Children’s Attributions and Perceptions Scale
CBCL: Child Behavior Checklist
CBT: Cognitive behavior therapy
CCT: Child-centered Counseling
CD-RISC: The Connor-Davidson Resilience scale
CGAS: Children's Global Assessment Scale
CPSS: Child PTSD Symptom Scale
C-PTAS: Child Post-Trauma Attitudes Scale
CSA: Childhood sexual assault
DSMB: Data Safety and Monitoring Board
GEE: Generalized estimating eqs.
IE: Independent Evaluator
LMM: Linear mixed-effects model
MASC: The Multidimensional Anxiety Scale for Children
MINI-KID: Mini International Neuropsychiatric Interview for Children and Adolescents
MRC: Medical Research Council
MSPSS: Multidimensional Scale of Perceived Social Support
NMR: Negative Mood Regulation
NSHW’s: Non-specialized primary care health workers (NSHW’s)
PDS: The Posttraumatic Stress Diagnostic Scale
PE: Prolonged exposure
PE-A: Prolonged Exposure for Adolescents
PESQ: Personal Experience Screening Questionnaire
PTCI: Posttraumatic Cognitions Inventory
PTSD: Posttraumatic Stress Disorder
RCT: Randomized control trials
SC: Supportive Counselling
SEM: Structural equations models
SOP: Standard operating procedure
STAI: State-Trait Anxiety Inventory
STAXI: The State-Trait Anger Expression Inventory
TF-CBT: Trauma-Focused Cognitive Behavioral Therapy
WCED: Western Cape Education Department
