# Supplementary material for: Prolonged exposure therapy and supportive counselling for posttraumatic stress disorder in adolescents in a community-based sample, including experiences of stakeholders: study protocol for a comparative randomized controlled trial using task-shifting
Source: BMC Psychiatry. 2018 Sep 6;18:288. doi: 10.1186/s12888-018-1873-x (PMC6127988; doi:10.1186/s12888-018-1873-x)
Supplement: Supplementary file 1 — Topic guides and interview schedules for the different qualitative groups, Written standard operating procedures. (DOCX 84 kb) [file 12888_2018_1873_MOESM1_ESM.docx]

**Additional File 1** - **Topic guides and interview schedules for the different qualitative groups**

**Focus Group Introduction Guideline:**

Thanks for agreeing to be part of this focus group. We appreciate your willingness to participate.

I have been asked to conduct focus groups to find out about the experience of people who have taken part in JR study so that we can figure out if it will be helpful for other people to provide this service to the community at large.

Firstly, I would like to go through 4 important things:

**Participation:** I would like everybody to participate. I may call on you if I haven’t heard from you in a while. Speak to each other! I want you to do the talking, not me.

**Honesty:** there are no right or wrong answers. People who survive a plane crash all have different ways of looking at the experience. So feel free to agree or disagree. I want to hear as many different opinions possible.

**Confidentiality and respect:** let us all agree to keep what happens tonight between us. Treat each other with respect (take turns, try to not interrupt) so that folks can feel comfortable sharing when sensitive issues arise.

**Recording:** we want to remember everything you say. The co-facilitator keeps written notes in case the recording fails, and the recording is for where he can’t get it all written down in time you’re your name gets used in the recording, we will make up a false name for the written copy of the session so that you can remain anonymous. We won’t identify anybody by name in our report – you will remain anonymous.

**Practical:** the co-facilitator here plays an important role to keep the show on the road. He will indicate the half-way mark for a bathroom break, deal with any interruptions, and also provide you with additional support and remove you from the group should it be needed.

**Table S4: Standard Operating Procedures for Adolescents**

**NB: When arranging appointment times, consider your safety and the safety of the participants: Interviews can take an hour. Be sure the interview does not end too late, preferably before dark.**

| **Step** | **Description** | | | | | | | | | **Initial** | **Date** |
| --- | --- | --- | --- | --- | --- | --- | --- | --- | --- | --- | --- |
| **Set up the information session** | | | | | | | | | | | |
| 1 | Retrieve contact details from database of adolescents who have already consented to participate in RCT over last year  Participant ID: ____________________________________________  Contact nr: _______________________________________________ | | | | | | | | |  |  |
| 2a | Phone parent to set up information session according to ***Table 4a***. First attempt. | | | | | | | | |  |  |
|  | Agreed to information session | | Call back later | | Left voicemail | | Nr works but no voicemail | Nr does not work, sent letter to school | |  |  |
| 2b | Phone parent to set up information session. Second attempt. | | | | | | | | |  |  |
|  | Agreed to information session | | Call back later | | Left voicemail | | Nr works but no voicemail | Nr does not work, sent letter to school | |  |  |
| 2c | Phone parent to set up information session. Final attempt. | | | | | | | | |  |  |
|  | Agreed to information session | | Call back later | | Left voicemail | | Nr works but no voicemail | Nr does not work, sent letter to school | |  |  |
| 2d | Follow up on letter to school. | | | | | | | | |  |  |
|  | Agreed to information session.  New contact details:___________________ | | | | | | | Lost to follow up. | |  |  |
| 3a | Arrange venue at school (or other suitable venue based on parents work commitments) for information session.  Venue: __________________________________________________  Contact person: ___________________________________________  Contact Nr: _______________________________________________ | | | | | | | | |  |  |
| 3b | **Should the school be unavailable as a venue for the information letter or a means to deliver the letter/the parent be unavailable to sign the ICF in person, the following procedure will be followed (hereby replacing steps 5 – 12):** | | | | | | | | |  |  |
|  | 1 | **Reason for alternative ICF procedure: ____________________** | | | | | | | |  |  |
|  | 2 | **Information letter and ICF read over telephone in suitable language** | | | | | | | |  |  |
|  | 3 | **Encouraged to ask questions and questions answered** | | | | | | | |  |  |
|  | 4 | **Telephonic consent obtained** | | | | | | | |  |  |
|  | 5 | **Date set for interview** | | | | | | | |  |  |
|  | 6 | **Transport arranged for interview** | | | | | | | |  |  |
|  | 7 | **Obtain teenager assent before interview starts** | | | | | | | |  |  |
|  | 8 | **Provide ICF to teenager before leaves, stressing the importance of the letter** | | | | | | | |  |  |
|  | 9 | **When set up the date for the focus group remind teenager to bring consent form** | | | | | | | |  |  |
|  | 10 | **Remind teenager and parent the day before and day of via sms** | | | | | | | |  |  |
|  | 11 | **Driver viewed signed consent form before allowing child into the car. No signed consent form = no focus group participation** | | | | | | | |  |  |
|  | 12 | **Signed consent form collected and filed** | | | | | | | |  |  |
|  | 13 | **Copy of consent form given to teenager to return home** | | | | | | | |  |  |
| 3c | **Should consent form become lost follow the following procedure:** | | | | | | | | |  |  |
|  | 1 | **Phone parent and remind them of the consent form. Ask them to send it to school.** | | | | | | | |  |  |
|  | 2 | **Phone teenager and remind them of the consent form. Ask them to bring it to school.** | | | | | | | |  |  |
|  | 3 | **Send a reminder sms to parent and teenager:** | | | | | | | |  |  |
|  |  | **Once** | | **Twice** | | **Thrice** | | | **Last time** |  |  |
|  | 4 | **Arrange a home visit to collect the form.** | | | | | | | |  |  |
|  | 5 | **Should the parent withdraw telephonic consent or the teenager withdraw assent, their individual data will no longer be used. No signed consent form, means no participation in focus group.** | | | | | | | |  |  |
| 4 | Send a reminder sms 24 hours in advance. | | | | | | | | |  |  |
| **Obtain informed consent** | | | | | | | | | | | |
| 5 | Discuss information letter with adolescent and parent together. | | | | | | | | |  |  |
| 6 | Both parties asked questions and received satisfactory answers. | | | | | | | | |  |  |
| 7 | Potential participant given time to consider proposition. | | | | | | | | |  |  |
| 8 | Parent signed consent for semi-structured interview (if no, stop here) | | | | | | | | |  |  |
| 9 | Parent signed consent for focus group (if no, stop at step 28) | | | | | | | | |  |  |
| 10 | Adolescent signed assent for semi-structured interview (if no, stop here) | | | | | | | | |  |  |
| 11 | Adolescent signed assent for focus group (if no, stop at step 28) | | | | | | | | |  |  |
| 12 | Parent receive compensation for time/inconvenience/expenses incurred to attend the session | | | | | | | | |  |  |
| **Prepare for the interview** | | | | | | | | | | | |
| 13 | Set a date for the semi-structured interview. Date: ________________ | | | | | | | | |  |  |
| 14 | Book a venue at school for the interview.  Venue: __________________________________________________  Contact person: ___________________________________________  Contact Nr: _______________________________________________ | | | | | | | | |  |  |
| 15 | Make a reminder phone call 24 hours in advance for the interview. | | | | | | | | |  |  |
| 16 | Make sure recording device is charged | | | | | | | | |  |  |
| 17 | Arrive at venue 20 minutes early to check availability | | | | | | | | |  |  |
| **Conduct the interview** | | | | | | | | | | | |
| 18 | Introductions and rules | | | | | | | | |  |  |
| 19 | Switch on the recording device | | | | | | | | |  |  |
| 20 | Conduct the interview according to ***Table 4b*** | | | | | | | | |  |  |
| 21 | Hand out incentive (sweets to the value of R20) and farewell | | | | | | | | |  |  |
| 22 | Make additional comments and field notes on the audio-recorder before switching it off. | | | | | | | | |  |  |
| **Prepare interview for transcription and coding** | | | | | | | | | | | |
| 23 | Clean up the venue | | | | | | | | |  |  |
| 24 | Upload recording onto Dropbox: for transcriber 1 | | | | | | | | |  |  |
| 25 | Upload recording onto OneDrive: for transcriber 2 | | | | | | | | |  |  |
| 26 | Receive transcription 1 | | | | | | | | |  |  |
| 27 | Receive transcription 2 | | | | | | | | |  |  |
| 28 | Reconcile any differences | | | | | | | | |  |  |
| 29 | Remove identifiable information | | | | | | | | |  |  |
| 30 | Start coding process according to ***Appendix 9*** | | | | | | | | |  |  |
| **Prepare for the focus group** | | | | | | | | | | | |
| 31 | Allocate to focus group PE-A or focus group SC, if enough participants, place in gender specific group. | | | | | | | | |  |  |
| 31 | Choose focus group date in collaboration with co-facilitator.  Date: ___________________________________________________ | | | | | | | | |  |  |
| 32 | Confirm date and transport logistics with participant.  Collection point: ___________________________________________  Collection time: ___________________________________________  Contact nr: _______________________________________________ | | | | | | | | |  |  |
| 33 | Book focus group transport with times and collection points according to Appendix 10. | | | | | | | | |  |  |
| 34 | Reminder sms 24 hours in advance | | | | | | | | |  |  |
| **On the day of the focus group** | | | | | | | | | | | |
| 35 | Remind transport of times and collection points. | | | | | | | | |  |  |
| 36 | Check that the venue is clean and open. | | | | | | | | |  |  |
| 37 | Set up chairs in circle | | | | | | | | |  |  |
| 38 | Set out refreshments (mints, glasses, water) | | | | | | | | |  |  |
| 39 | Prepare name tags and pens | | | | | | | | |  |  |
| 40 | Put up signs to lead to the venue | | | | | | | | |  |  |
| 41 | Prepare the incentive packs (sweets to the value of R20) | | | | | | | | |  |  |
| **Conduct the focus group** | | | | | | | | | | | |
| 42 | PI welcomes the group and makes introductions | | | | | | | | |  |  |
| 43 | When PI leaves, hand out name tags and make up code name | | | | | | | | |  |  |
| 44 | Explain introduction and rules according to **Focus Group Introduction Guideline** | | | | | | | | |  |  |
| 45 | Switch on recorder | | | | | | | | |  |  |
| 46 | Work through topic guide in ***Table 4b*** | | | | | | | | |  |  |
| 47 | Hand out incentive and say goodbye | | | | | | | | |  |  |
| 48 | While recorder is on, debrief thoughts with co-facilitator. Once all thoughts are exhausted switch the device off. | | | | | | | | |  |  |
| **Prepare for transcription and coding** | | | | | | | | | | | |
| 49 | Clean up the venue | | | | | | | | |  |  |
| 50 | Upload recording onto Dropbox: for transcriber 1 | | | | | | | | |  |  |
| 51 | Upload recording onto OneDrive: for transcriber 2 | | | | | | | | |  |  |
| 52 | Receive transcription 1 | | | | | | | | |  |  |
| 53 | Receive transcription 2 | | | | | | | | |  |  |
| 54 | Reconcile any differences | | | | | | | | |  |  |
| 55 | Remove identifiable information | | | | | | | | |  |  |
| 56 | Start coding process according to ***Appendix 9*** | | | | | | | | |  |  |

**Table S4a: Initial Telephonic Contact with Adolescent Parent**

| Good day. My name is … I am calling from Stellenbosch University with regards to the PTSD study that your child has been involved in. Is now a good time to talk? | | |
| --- | --- | --- |
| Great! I am calling today to ask your permission for your child to participate in an interview and a focus group. In this one-on-one interview and focus group, I will be inviting your child to share his/her experiences with me to find out what he/she liked/disliked, what helped, what was difficult etc. while participating in the PTSD counselling.  During the school holidays, I would like to   1. Arrange for transport (at no cost to you) to collect your child at home, bring him/her to Stellenbosch University medical campus, and then return him/her home. 2. At the university in a private office, your child and I (a clinical psychologist) will have a 60 minute audio-recorded interview. 3. On a different occasion, there will be another meeting at the university. I will arrange transport again. At this meeting, your child will form part of a group discussion with other children who participated in the study discussing their experiences. This group session will be 90 minutes long, facilitated by myself, and again be audio-recorded. 4. On both occasions your child will receive a small snack-pack and a R20 voucher.   Is your child currently at school? We can set up a date to discuss the form and sign it together at school. Alternatively, I can send the letter to school and then you send it to me signed with your child when I see him/her for the interview.  If it is impossible to meet at the school or deliver the letter via school, I would like to use this telephonic conversation to answer all of the questions you may have, and then send the letter home with your child after our first interview. You can then read the letter at home and send it (signed) with your child when he/she comes to the focus group. Alternatively, you can give me your email address and I can send the letter to you in that way.  Are you agreeable to this?  Great! Let me read the letter to you.  Now that you have granted telephonic consent, I would like to set up a date for the interview with your child where I will provide him/her with an opportunity to provide their written assent, and send them home with a letter to sign (your written consent). | When would be a suitable time to call? | May I send you an information letter via your school? |

**Table S4b: Adolescent interview schedule/ Adolescent Focus group topic guide**

| **Domain** | **Question** | **Prompts** |
| --- | --- | --- |
| **Need for treatment** | 1. How did you come to participate in this study? Tell me about the internal process that made you agree to accept treatment. | When did you move from the “I can sort it out myself” to the “I need help” phase? What resources did you consider? Where did you try to get help before? |
| **Acceptability (Like/dislike):** | 1. What do you like/dislike about the therapeutic process? What was helpful/unhelpful? | Have you done something like this before? Was it what you expected? What was your favourite moment in therapy? What was the hardest thing to do? |
|  | 1. What about the counsellor? What did you like/dislike, was helpful/unhelpful? | What made it different to speak to a counsellor than somebody at home/a friend? |
| **Feasibility (barriers/ facilitators):** | 1. How did you overcome barriers to accessing treatment/stigma? | What really stood in your way to getting help? Transport? To go to the clinic must skip school? |
|  | 1. What do you think prevents others from accessing this service at school? | Are there other kids who could benefit from something like this? |
|  | 1. What was it like to receive treatment at school? | Flack from friends or teachers? What did you say when you came for your appointment? Would it be different if it happened at the clinic? |
| **Impact (pros and cons):** | 1. What was the outcome of treatment? | In what way did it help you? How are you different now? |
|  | 1. What are you still implementing? | What did you learn? What was specifically helpful? What other domains in your life has this intervention helped? |
|  | 1. Would you recommend this process to a friend going through a hard time? | Why? What would classify as a hard time? How would you go about recommending it? Why would this help them? |
| **Suggestions:** | 1. What suggestions would you make to ensure that the program is even better? | If you had to be in charge of this program and make it available to all teens, what would you do to make it better? |

**Table S5: Standard Operating Procedures for Nurses**

| **Step** | **Description** | | | | | **Initial** | **Date** |
| --- | --- | --- | --- | --- | --- | --- | --- |
| **Set up the information session** | | | | | | | |
| 1 | Retrieve contact details from database of nurses who have been involved in project over last year  Participant ID: ____________________________________________  Contact nr: _______________________________________________ | | | | |  |  |
| 2a* | Phone participant to set up information session according to ***Table 5a***. First attempt. | | | | |  |  |
|  | Agreed to information session | Call back later | Left voicemail | Nr works but no voicemail | Nr does not work, sent an email |  |  |
| 2b | Phone participant to set up information session. Second attempt. | | | | |  |  |
|  | Agreed to information session | Call back later | Left voicemail | Nr works but no voicemail | Nr does not work, sent an email |  |  |
| 2c | Phone participant to set up information session. Final attempt. | | | | |  |  |
|  | Agreed to information session | Call back later | Left voicemail | Nr works but no voicemail | Nr does not work, sent an email |  |  |
| 2d | Follow up on email | | | | |  |  |
|  | Agreed to information session.  New contact details:___________________ | | | | Lost to follow up. |  |  |
| 3 | Arrange venue at school for information session.  Venue: __________________________________________________  Contact person: ___________________________________________  Contact Nr: _______________________________________________ | | | | |  |  |
| 4 | Send a reminder sms 24 hours in advance. | | | | |  |  |
| **Obtain informed consent** | | | | | | | |
| 5 | Discuss information letter with participant. | | | | |  |  |
| 6 | Answer the participant’s questions. | | | | |  |  |
| 7 | Potential participant given time to consider proposition. | | | | |  |  |
| 8 | Participant signed consent for focus group (if no, stop here). | | | | |  |  |
| 9 | Ask participant to highlight 10 days between 1 August and 30 September where they could be available for a focus group. | | | | |  |  |
| **Prepare for the focus group** | | | | | | | |
| 10 | Choose focus group date in collaboration with co-facilitator.  Date: ___________________________________________________ | | | | |  |  |
| 11 | Confirm date and transport logistics with participant.  Collection point: ___________________________________________  Collection time: ___________________________________________  Contact nr: _______________________________________________ | | | | |  |  |
| 12 | Book focus group transport with times and collection points. | | | | |  |  |
| 13 | Reminder sms 24 hours in advance | | | | |  |  |
| **On the day of the focus group** | | | | | | | |
| 14 | Remind transport of times and collection points. | | | | |  |  |
| 15 | Check that the venue is clean and open. | | | | |  |  |
| 16 | Set up chairs in circle | | | | |  |  |
| 17 | Set out refreshments (mints, glasses, water) | | | | |  |  |
| 18 | Prepare name tags and pens | | | | |  |  |
| 19 | Put up signs to lead to the venue | | | | |  |  |
| 20 | Prepare the incentive packs (R50 Woolworths voucher) | | | | |  |  |
| **Conduct the focus group** | | | | | | | |
| 21 | PI welcomes the group and makes introductions: | | | | |  |  |
| 22 | When PI leaves, hand out name tags and make up code name | | | | |  |  |
| 23 | Explain introduction and rules according to ***Focus Group Introduction Guideline*** | | | | |  |  |
| 24 | Switch on recorder | | | | |  |  |
| 25 | Hand out open ended questionnaire grid ***Table 5c*** | | | | |  |  |
| 26 | Work through topic guide ***Table 5b*** | | | | |  |  |
| 27 | Arrange method for member checking: e.g. via email with due date | | | | |  |  |
| 28 | Hand out incentive (remember to sign) and say goodbye | | | | |  |  |
| 29 | While recorder is on, debrief thoughts with moderator. Once all thoughts are exhausted switch the device off. | | | | |  |  |
| **Prepare for transcription and coding** | | | | | | | |
| 30 | Clean up the venue | | | | |  |  |
| 31 | Upload recording onto Dropbox: for transcriber 1 | | | | |  |  |
| 32 | Upload recording onto OneDrive: for transcriber 2 | | | | |  |  |
| 33 | Receive transcription 1 | | | | |  |  |
| 34 | Receive transcription 2 | | | | |  |  |
| 35 | Reconcile any differences | | | | |  |  |
| 36 | Remove identifiable information | | | | |  |  |
| 37 | Email the transcription to participant according to arrangements in step 27. | | | | |  |  |
| 38 | Make final changes according to participant’s feedback | | | | |  |  |
| 39 | Commence with coding and analysis according to ***Appendix 9*** | | | | |  |  |

**Table S5a: Initial Telephonic Contact**

| Good day. My name is … I am calling from Stellenbosch University with regards to the PTSD study that you have been involved in. Is now a good time to talk? | | |
| --- | --- | --- |
| Great! I am calling today to ask you to share your experiences with me: about what it was like to participate in the PTSD study you participated in last year. We hope to use this information to inform project scalability: in other words – would it be possible to replicate this project in a larger scale to serve the community. Your inputs would be very valuable!  Should you choose to participate I will set up a date with some of the other nurses that you may already know to discuss your experiences in a focus group at the University. I will ask 10 questions and it should take about 90 minutes. To remember what you say, I will make an audio-recording. Don’t worry, the PI will not be in the sessions with us and he won’t have access to the recordings. All the information he gets will be de-identified meaning that he won’t know who said what.  When can you and I meet to give you more information, and space to ask questions? Would you like to meet with me at Tygerberg campus? | When would be a suitable time to call? | May I send you an information letter via email? |

**Table S5b: Nurse topic guide**

| **Domain** | **Question** | **Prompts** |
| --- | --- | --- |
| **Activities** | 1. I will give you 5 minutes to jot down your ideas in the handout. We will use this as part of our discussion. 2. What was your experience of the many different activities you were involved in? *Refer to handout and their notes.* | See handout ***Table 5c*** Like, dislike  Barriers, facilitators  Pros, cons  Training and supervision  PE-A and SC |
|  | 1. What was it like to counsel traumatized teenagers? | What helped you to cope? What was difficult? What surprised you? |
| **Acceptability (Like/dislike):** | 1. What intervention (parts of the intervention) did you prefer? Why? | Supportive counselling?  Prolonged exposure?  Recruitment?  Going to schools? |
|  | 1. What was it like to receive group supervision? Especially if you did not get to provide both treatments? | How was it different?  What was hard? What were you relieved about? |
| **Feasibility (barriers/ facilitators):** | 1. What are the rewards and challenges of being part of this study? | What was your highlight? Lowlight? |
|  | 1. How did a contact person at school influence the success of this process? | How can one identify such a person? What did the person do? |
|  | 1. What would make you feel more equipped to do these interventions? | Manual? More freedom?  Practice? |
| **Impact (pros and cons):** | 1. Why would you (not) continue with task shifting as a future career? | What kind of feedback do you get from others when you tell them you are part of this project? |
|  | 1. What are you still implementing from your learning during this project? | How did you grow? How are you different now? |
| **Suggestions:** | 1. What suggestions would you make to ensure that this project’s success and sustainability? | If you were in charge, how would you make it better? |

**Table S5c: Questionnaire for Nurses/Counsellors (handout)**

|  | **Acceptability** | | **Feasibility** | | **Impact** | |
| --- | --- | --- | --- | --- | --- | --- |
|  | What did you like about | What did you dislike about | What barriers hindered success in | What factors enabled success in | What are the advantages of | What are the disadvantages |
| **The training** |  |  |  |  |  |  |
| **Prolonged exposure therapy** |  |  |  |  |  |  |
| **Supportive counselling** |  |  |  |  |  |  |
| **Supervision** |  |  |  |  |  |  |

**Table S6: Standard Operating Procedures for Teachers**

| **Step** | **Description** | | | | | **Initial** | | **Date** | |  |
| --- | --- | --- | --- | --- | --- | --- | --- | --- | --- | --- |
| **Set up the information session** | | | | | | | | | |  |
| 1 | Retrieve contact details from database of school contact persons who have been involved in project over last year.  Participant ID: ____________________________________________  Contact nr: _______________________________________________ | | | | | |  | |  | |
| 2a* | Phone participant to set up information session according to ***Table 6a***. First attempt. | | | | | |  | |  | |
|  | Agreed to information session | Call back later | Left voicemail | Nr works but no voicemail | Nr does not work, sent letter to school | |  | |  | |
| 2b | Phone participant to set up information session. Second attempt. | | | | | |  | |  | |
|  | Agreed to information session | Call back later | Left voicemail | Nr works but no voicemail | Nr does not work, sent letter to school | |  | |  | |
| 2c | Phone participant to set up information session. Final attempt. | | | | | |  | |  | |
|  | Agreed to information session | Call back later | Left voicemail | Nr works but no voicemail | Nr does not work, sent letter to school | |  | |  | |
| 2d | Follow up on letter to school. | | | | | |  | |  | |
|  | Agreed to information session.  New contact details:___________________ | | | | Lost to follow up. | |  | |  | |
| 3 | Arrange venue at school for information session.  Venue: __________________________________________________  Contact person: ___________________________________________  Contact Nr: _______________________________________________ | | | | | |  | |  | |
| 4 | Send a reminder sms 24 hours in advance. | | | | | |  | |  | |
| **Obtain informed consent** | | | | | | | | | | |
| 5 | Discuss information letter with participant. | | | | | |  | |  | |
| 6 | Answer the participant’s questions. | | | | | |  | |  | |
| 7 | Potential participant given time to consider proposition. | | | | | |  | |  | |
| 8 | Participant signed consent for focus group (if no, stop here). | | | | | |  | |  | |
| 9 | Ask participant to highlight 10 days between 1 August and 30 September where they could be available for a focus group. | | | | | |  | |  | |
| **Prepare for the focus group** | | | | | | | | | | |
| 10 | Choose focus group date in collaboration with co-facilitator.  Date: ___________________________________________________ | | | | | |  | |  | |
| 11 | Confirm date and transport logistics with participant.  Collection point: ___________________________________________  Collection time: ___________________________________________  Contact nr: _______________________________________________ | | | | | |  | |  | |
| 12 | Book focus group transport with times and collection points. | | | | | |  | |  | |
| 13 | Reminder sms 24 hours in advance | | | | | |  | |  | |
| **On the day of the focus group** | | | | | | | | | | |
| 14 | Remind transport of times and collection points. | | | | | |  | |  | |
| 15 | Check that the venue is clean and open. | | | | | |  | |  | |
| 16 | Set up chairs in circle | | | | | |  | |  | |
| 17 | Set out refreshments (mints, glasses, water) | | | | | |  | |  | |
| 18 | Prepare name tags and pens | | | | | |  | |  | |
| 19 | Put up signs to lead to the venue | | | | | |  | |  | |
| 20 | Prepare the incentive packs (R50 Woolworths voucher) | | | | | |  | |  | |
| **Conduct the focus group** | | | | | | | | | | |
| 21 | PI welcomes the group and makes introductions | | | | | |  | |  | |
| 22 | When PI leaves, hand out name tags and make up code name | | | | | |  | |  | |
| 23 | Explain introduction and rules according to ***Focus Group Introduction Guideline*** | | | | | |  | |  | |
| 24 | Switch on recorder | | | | | |  | |  | |
| 25 | Work through topic guide according to ***Table 6b*** | | | | | |  | |  | |
| 26 | Hand out incentive (remember to sign) and say goodbye | | | | | |  | |  | |
| 27 | While recorder is on, debrief thoughts with moderator. Once all thoughts are exhausted switch the device off. | | | | | |  | |  | |
| 28 |  | | | | | |  | |  | |
| **Prepare for transcription and coding** | | | | | | | | | | |
| 29 | Clean up the venue | | | | | |  | |  | |
| 30 | Upload recording onto Dropbox: for transcriber 1 | | | | | |  | |  | |
| 31 | Upload recording onto OneDrive: for transcriber 2 | | | | | |  | |  | |
| 32 | Receive transcription 1 | | | | | |  | |  | |
| 33 | Receive transcription 2 | | | | | |  | |  | |
| 34 | Reconcile any differences | | | | | |  | |  | |
| 35 | Remove identifiable information | | | | | |  | |  | |
| 36 | Commence with thematic analysis according to ***Appendix 9*** | | | | | |  | |  | |

**Table S6a: Initial Telephonic Contact**

| Good day. My name is … I am calling from Stellenbosch University with regards to the PTSD study that you have been involved in. Is now a good time to talk? | | |
| --- | --- | --- |
| Great! I am calling today to ask you to share your experiences with me: about what it was like to participate in the PTSD study you participated in last year. We hope to use this information to inform project scalability: in other words – would it be possible to replicate this project in a larger scale to serve the community. Your inputs would be very valuable!  Should you choose to participate I will set up a date with teachers from other schools that took part in the study to discuss your experiences in a focus group at the University. I will ask 10 questions and it should take about 90 minutes. To remember what you say, I will make an audio-recording. Don’t worry, PI will not be in the sessions with us and he won’t have access to the recordings. All the information he gets will be de-identified meaning that he won’t know who said what.  When can you and I meet, to give you more information and space to ask questions? I can arrange a room for us at your school. | When would be a suitable time to call? | May I send you an information letter via your school/email? |

**Table S6b: Teacher topic guide**

| **Domain** | **Question** | **Prompts** |
| --- | --- | --- |
| **Activities** | 1. How did you become involved in this project? | What do you know about this project? |
|  | 1. What was your role in this project? | Did you choose it? Were you assigned to this role? |
| **Acceptability (Like/dislike):** | 1. What did you like/dislike about this role? | Popular? Help the children? Extra work and pressure? Resented being forced? |
|  | 1. What did you think about the project when you first heard about it? | A good idea? Bad idea? |
|  | 1. How did your opinion change/stay the same during the course of this project? |  |
| **Feasibility (barriers/ facilitators):** | 1. What do you think made the recruitment at school un/successful? | How many scholars from your school? Should there have been more? Why? How do you identify those children? What would have made it possible for them to get this kind of help? |
|  | 1. What are the practical implications and challenges of arranging for scholars to receive treatment at school? | Venues? Time? Contact with the team? |
| **Impact (pros and cons):** | 1. What effects could you see as a result of the intervention? | Did it help or not? Stigma? Interaction with other scholars? |
| **Suggestions:** | 1. Why would you dis/encourage mental health interventions at your school in the future? | And for other schools? For your own child? |
|  | 1. What suggestions or recommendations would you make to ensure this projects’ success and sustainability? | If you were in charge of the department of education, would you recommend that this project gets rolled out on a larger scale? What changes would you make? |

**Table S7: SOP for Data Coding and Thematic Analysis**

| **Step** | **Description** | **Initial** | **Date** |
| --- | --- | --- | --- |
| **Coding draft 1: begin coding** | | | |
| 1 | Read through transcript* while listening to audio recording |  |  |
| 2 | Identify any new themes and add to the coding tree |  |  |
| 3 | Code paragraphs according to thematic coding scheme |  |  |
| 4 | Send to researcher |  |  |
| **Coding draft 2: identify missing themes** | | | |
| 5 | Researcher read through transcript while listening to audio-recording if allowed (get immersed in the data) |  |  |
| 6 | Researcher identify more themes |  |  |
| 7 | Send to coder |  |  |
| 8 | Coder add those themes to coding |  |  |
| **Analysis draft 1: put it together** | | | |
| 9 | Thematic analysis on Atlas.ti: what do the themes mean?** |  |  |
| **Analysis draft 2: highlight contradictions** | | | |
| 10 | Read through transcripts to find contradictions to findings |  |  |
| 11 | Re-work and integrate in thematic analysis |  |  |
| 12 | Send to researcher |  |  |
| **Analysis draft 3: identify missing depth** | | | |
| 13 | Researcher to read through transcripts and compare with analysis to see what is missing |  |  |
| 14 | Re-work and integrate |  |  |
| 15 | Analysis 3 complete |  |  |

*Throughout, make notes and provide feedback to psychologist to improve data depth and quality

**Best done by researcher, but deemed sufficient to be done by coder for purposes of this study

**Table S8: SOP for Transport Arrangements of Participants to SU Medical Campus**

| **Step** | **Description** | **Initial** | **Date** |
| --- | --- | --- | --- |
| **Make sure the transport service complies with criteria for departmental approval** | | | |
| 1 | The service complies with departmental approval |  |  |
| 2 | The driver has a licence |  |  |
| 3 | The driver has a permit to travel groups of people, including children |  |  |
| 4 | The driver has personal insurance |  |  |
| 5 | The vehicle is insured |  |  |
